# Is exposure to hydrocarbons associated with chronic kidney disease in young Nigerians? A case–control study

**DOI:** 10.3389/fneph.2022.1010080

**Published:** 2022-12-16

**Authors:** Ogochukwu Chinedum Okoye, Nyemike Awunor

**Affiliations:** ^1^ Department of Medicine, Delta State University, Abraka, Nigeria; ^2^ Department of Internal Medicine, Delta State University Teaching Hospital, Oghara, Nigeria; ^3^ Department of Community Medicine, Delta State University, Abraka, Nigeria; ^4^ Department of Community Medicine, Delta State University Teaching Hospital, Oghara, Nigeria

**Keywords:** chronic kidney disease, CGN, exposure, hydrocarbon, petrochemicals, Niger Delta, young adults

## Abstract

**Introduction:**

Although environmental exposure to hydrocarbons has been linked to non-communicable diseases, its association with chronic kidney disease (CKD) is still an emerging area. Epidemiological studies associating CKD with prolonged exposure to hydrocarbons have mostly focused on occupational exposure, with fewer studies on environmental exposure from residing in contaminated areas. The aim of this study was to determine any association between long-term exposure to petrochemical products and the risk of CKD by comparing the residence and occupational history of young patients with CKD and non-CKD controls.

**Materials and methods:**

A case–control study of 74 cases and 74 age- and sex-matched non-CKD controls was carried out. Cases were patients with CKD who were aged 18–44 years and diagnosed with suspected chronic glomerulonephritis (CGN). Patients were recruited from an outpatient nephrology clinic and medical wards. Patients with CKD from traditional causes were excluded. Data were collected using a pre-tested structured questionnaire adapted from the WHO STEPwise approach to the non-communicable disease risk factor surveillance (STEPS) instrument. To assess exposure, a detailed work history and all residential addresses where the patients have lived for at least 5 years were recorded. ‘Exposed’ status was regarded as long-term residence in a known oil-polluted area and jobs involving crude oil exploration, processing, transportation and sales, and cleanup of crude oil hazards. Absence of a history of chronic exposure or any form of exposure was regarded as ‘less exposed’.

**Results:**

There were 52 (70.3%) cases categorized as exposed, compared with 21 (28.4%) controls (*p *< 0.001). There were 34 (45.9%) cases born near petrochemical refineries and plants, compared with 11 (14.9%) controls (*p* ≤ 0.001). There were 34 (45.9%) cases residing near petrochemical refineries and plants, compared with 8 (10.9%) controls (*p* ≤ 0.001). When asked ‘Do you think you have been significantly exposed to crude oil?’, 15 (20.3%) cases and 2 (2.7%) controls answered ‘yes’ (*p* ≤ 0.001).

**Conclusion:**

Our findings suggest an association between exposure to petrochemicals and CKD in young Nigerians diagnosed with suspected CGN. Exposure is significantly associated with a higher mean age, waist circumference, and blood sugar levels; however, other traditional risk factors for CKD were not considerably more prevalent in this unique patient population. These findings should prompt more emphasis on occupational history, residential history, and other relevant environmental exposures in the assessment of patients at risk for CKD.

## Introduction

Environmental exposures are linked with 24% of deaths globally, mainly from non-communicable diseases (NCDs), and the burden is higher in low- and middle-income countries, where environmental health policies are usually not effectively enforced and health literacy is poor (1). Human exposure to petrochemical products has been linked to some NCDs, including cardiovascular disease, liver disease, hematological disorders, cancers, and congenital malformation (2); however, the association with chronic kidney disease (CKD) is still an emerging area. Although studies have associated some forms of CKD with prolonged human exposure to hydrocarbons, most of the studies were epidemiological studies focused on occupational exposure, with few studies on environmental exposure from residing in contaminated areas (3). Yet the evidence of a causal association between exposure to petrochemical products and CKD remains conflicting.

Chronic kidney disease is an NCD that is associated with significant mortality and morbidity globally, but, unfortunately, in contrast to other NCDs, it has not received adequate attention from the public health community. In low- and middle-income countries, CKD affects young and economically productive youths; however, in western countries, CKD is a disease that affects the elderly (4). The common traditional risk factors for CKD, such as hypertension and diabetes, do not sufficiently account for the higher burden of CKD in the young population, and so the search for newer risk factors, such as environmental exposures and genetic predispositions, is justified.

The direct mechanism for kidney injury following exposure to hydrocarbons is linked to developmental physiological disruption and oxidative stress. Peroxidation of lipids induces cellular damage and inflammation, whereas oxidative stress alters the endothelial relaxant nitric oxide, thus promoting vasoconstriction, platelet adhesion, and release of cytokines (5). Furthermore, excessive oxidative stress has been shown to alter the podocyte skeleton, leading to albuminuria, podocyte loss, and tubular injury, which are markers of progressive glomerular disease. The vulnerability of an individual to the toxicity of hydrocarbons, and most nephrotoxins, usually depends on the physical characteristics of the toxin, dose, duration of exposure, pre-existing nutritional state, comorbidities, socioeconomic status, and negative habits; however, even the smallest amount of exposure for prolonged periods can have deleterious health effects (2).

The Niger Delta region of Nigeria accounts for a significant proportion of crude oil production in West Africa, and some communities located near petrochemical refineries and plants have suffered significant environmental degradation and potential health risks (6). These negative environmental and health impacts are due to the toxic effects of polycyclic aromatic hydrocarbons, benzenes, and other toxic gases that contaminate the land, water, and air. Medical professionals in the Niger Delta speculate that residents of this region may constitute the bulk of patients with specific diseases, such as respiratory diseases, congenital malformations, cancers, and CKD, seen in health institutions; however, the evidence is lacking. Residing near a petrochemical plant may lead to long-term exposure to high levels of hydrocarbons and other contaminants that have been shown to be nephrotoxic in both animal and human studies (7–12).

Epidemiological evidence on the association between hydrocarbon exposure and CKD remains conflicting. Okoye reviewed the existing literature on the subject and identified 64 relevant articles, which were summarized in a review article (3). Notably, only a few studies originated from the Global South; for instance, only two African studies were included, and these were cross-sectional. Few studies investigated exposure resulting from residing close to petrochemical industries, oil rigs, or in communities that have experienced repeated oil spills, as is common in small communities in southern Nigeria. Overall, 64% of studies (including cohort and case–control studies and one meta-analysis) support an association between hydrocarbon exposure and kidney disease. Although there were methodological issues with some of the studies, the results of these earlier studies should not be ignored, but, rather, the studies should re-open the conversation on petrochemical- or hydrocarbon-related kidney disease.

Environmental contaminants mostly occur in combination, making it difficult to isolate any one toxin as the sole cause of kidney damage or adverse health effects. Furthermore, different types of hydrocarbons can individually cause multiple types of glomerulonephritis, suggesting that the histopathological pattern alone may not identify the exposure. Crude oil, for instance, is a combination of several potential toxins, and so the aim of epidemiological studies is not to identify the exact causative substance, but to establish a scientifically significant causal association between exposure and effect that will inform environmental health policies and interventions (13). Given the irreversible and progressive nature of CKD, the high cost of treatment, and adverse outcomes, especially in disadvantaged countries where regulations on environmental pollution are largely ineffective, further exploration of a link between CKD and a potentially modifiable environmental risk factor is essential.

The aim of this study was to determine any association between long-term exposure to petrochemical products and the risk for CKD by comparing the residence and occupational history of young patients with CKD with non-CKD controls.

## Materials and methods

### Study design

This was a case–control study of patients with CKD, using age- and sex-matched controls recruited from Delta State University Teaching Hospital, Oghara, in the Ethiope West Local Government Area of Delta State, Nigeria. This state-owned tertiary hospital serves as a referral center for patients with kidney disease in Delta State and other neighboring states. Ethics approval was obtained from the Health Research and Ethics Committee of Delta State University Teaching Hospital (reference HREC/PAN/2021/023/0335).

### Sample selection

Cases were new and old patients with CKD who were aged 18–44 years and diagnosed with suspected chronic glomerulonephritis (CGN). Cases were recruited from the outpatient nephrology clinic and inpatient service of Delta State University Teaching Hospital. Cases included old patients already on treatment. Diagnosis of suspected CGN is typically made when a patient with CKD presents with significant proteinuria with or without hematuria with active urine sediments, but without histological confirmation because of late presentation with advanced disease. Patients with CKD from established traditional causes, such as diabetes, hypertension, obstructive uropathy, autoimmune diseases, HIV infection, and hepatitis B or C infection, were excluded from this study. The controls in this study were sex- and age-matched (within 5 years) non-CKD patients recruited from the ophthalmology and ear, nose, and throat outpatient clinics. Patients with evidence of CKD, for example raised serum creatinine levels, reduced estimated glomerular filtration rate, or significant proteinuria, were excluded from the controls.

A minimum sample size of 74 cases and controls, respectively, was calculated using StatsDirect version 2.8.0. statistical software (StatsDirect Ltd, Wirral, UK) for case–control studies, assuming an exposure probability of 0.26 in the control group and an odds ratio (OR) of 3 (14). Eligible cases who met the criteria for inclusion were selected consecutively as they presented to the nephrology outpatient weekly clinic and inpatient service. Age- and sex-matched controls were recruited consecutively in weekly batches from the surgical outpatient clinics to match the number of cases recruited from the nephrology service. An updated list detailing the sex and age of cases recruited was used to guide the recruitment of controls as they presented to the specified weekly clinics. Cases and controls were recruited over a 6-month period until the sample size was achieved.

### Data collection

Data were collected using a pre-tested structured questionnaire adapted from the WHO STEPwise approach to non-communicable disease risk factor surveillance (STEPS) instrument (15) and a Benzene, Toluene, Ethylbenzene, and Xylene (BTEX) questionnaire obtained from the National Institute of Environmental Health Sciences’ (Durham, NC, USA) web page (16). The structured questionnaire included demographic variables, health status variables, relevant clinical history, and history of exposure to crude oil. Anthropometric variables and clinical measurements were recorded on a data sheet incorporated into the questionnaire. To assess exposure, detailed work histories and all residential addresses where the patients lived for at least 5 years were recorded (17). The self-reported current residence was confirmed with the patients’ recorded address in the hospital database. ‘Exposed’ status was regarded as long-term (at least 5 years) residence in a known oil/natural gas-situated community or having a job involving crude oil exploration, processing, transportation and sales, or cleanup of crude oil hazards. Absence of a history of chronic exposure or any form of exposure was regarded as ‘less exposed’.

Researchers and trained assistants administered the questionnaire during nephrology and ophthalmology outpatient clinic days. Cases were identified by the attending physician as they presented for consultation and were referred to the assistants for the informed consent process; only patients who gave consent were enrolled. Similarly, focal persons who were part of the nephrology team helped to identify eligible inpatients (cases only) and informed the interviewers, who then recruited cases, as above.

Basic laboratory tests, including serum creatinine and urinalysis, were carried out to screen and exclude controls with evidence of CKD.

### Data analysis

Data entry, cleanup, and management were performed using IBM SPSS Statistics Data Editor, version 27 (IBM Corporation, Armonk, NY, USA). For the purpose of analysis, exposure status was regarded as a dichotomous variable (i.e., exposed/less exposed). Exposure frequencies for cases and controls were presented as tables and as a bar chart. Chi-squared tests were used to test for any statistical association between the exposure status of cases and controls, and to compare the frequency of traditional risk factors of CKD between the exposed and non-exposed cases. Student’s *t*-tests were used for quantitative variables to ascertain any between-group statistical differences. Statistical significance was set at a *p*-value* *≤ 0.05 with a 95% CI.

## Results

### Demographics and clinical characteristics of cases and controls

There were 74 young (aged 18–44 years) patients with CKD and a diagnosis of suspected CGN were recruited as cases, and 74 non-CKD controls were recruited from surgical outpatient clinics (**Suppl. 1**). Females predominated in both groups (cases, 56.8%; controls, 55.4%). The mean (± SD) ages of cases and controls were 33.6 ± 7.8 years and 31.0 ± 8.8 years, respectively (*p = *0.056). There were 33 (44.6%) cases, compared with 3 (4.1%) controls, who reported a diagnosis of hypertension (*p* ≤ 0.001). Mean systolic blood pressure (SBP) and mean diastolic blood pressure (DBP) were higher in cases than in controls [mean SBP: cases, 126.2 ± 21.2 mmHg; controls, 116.3 ± 9.3 mmHg (*p = *0.001); mean DBP: cases, 79.3 ± 15.8 mmHg; controls, 75.0 ± 9.0 mmHg (*p = *0.470)]. The mean serum creatinine level in controls was 0.84 ± 0.13 mg/dl, and in cases was 1.45 (0.8) mg/dl (range 0.5–18.0 mg/dl). **Table 1** shows the demographics, clinical characteristics, and laboratory parameters of the cases and controls.

**Table 1:** Demographics, clinical characteristics, and laboratory parameters of cases and controls.

Characteristic	Cases (*N* = 74), *n* (%)	Control (*N* = 74), *n* (%)	*p*-value
Male	33(44.6)	32(43.2)	1.000
Female	41(55.4)	42(56.8)	
Smoking history	28(38.4)	34(45.9)	0.405
History of hypertension	33(44.6)	3(4.1)	**< 0.001**
History of dyslipidemia	3(4.1)	0(0.0)	0.245
Family history of kidney disease	3(4.1)	3(4.1)	1.000
History of sore throat	2(2.7)	1(1.4)	1.000
Use of herbal remedies	4(5.4)	0(0.0)	0.120
Use of skin lighteners	3(4.1)	4(5.4)	1.000
Use of NSAIDs	12(16.2)	4(5.4)	0.061
Use of herbicides/pesticides	10(13.5)	8(10.8)	0.802
**Clinical and laboratory measurement**	**Mean ± SD**	**Mean ± SD**	
Age (years)	31.0 ± 8.8	33.6 ± 7.8	0.056
BMI (kg/m^2^)	23.9 ± 4.5	26.2 ± 4.6	**0.003**
Waist circumference (cm)	86.1 ± 11.4	87.6 ± 11.4	0.548
SBP (mmHg)	126.2 ± 21.2	116.3 ± 9.3	**< 0.001**
DBP (mmHg)	79.3 ± 15.8	75.0 ± 9.0	0.470
Serum urea level (mg/dl)	63.7 ± 52.6	32.0 ± 8.0	**< 0.001**
Serum creatinine level (mg/dl)	3.2 ± 3.8	0.8 ± .0.1	**< 0.001**
Random blood sugar level(mg/dl)	103.4 ± 21.4	97.0 ± 18.0	0.057

BMI, body mass index; DBP, diastolic blood pressure; NSAID, non-steroidal anti-inflammatory drug; SBP, systolic blood pressure.

The distribution of the 74 cases based on CKD stage (calculated using the CKD-EPI creatinine equation) was as follows: stage 1, *n* = 11; stage 2, *n* = 11; stage 3, *n* = 9; stage 4 *n* = 9; and stage 5, *n* = 18. There was no association between stages of CKD and exposure status (χ^2^ = 2.583; *p* = 0.583).

### Exposure status of patients

**Figure 1:**
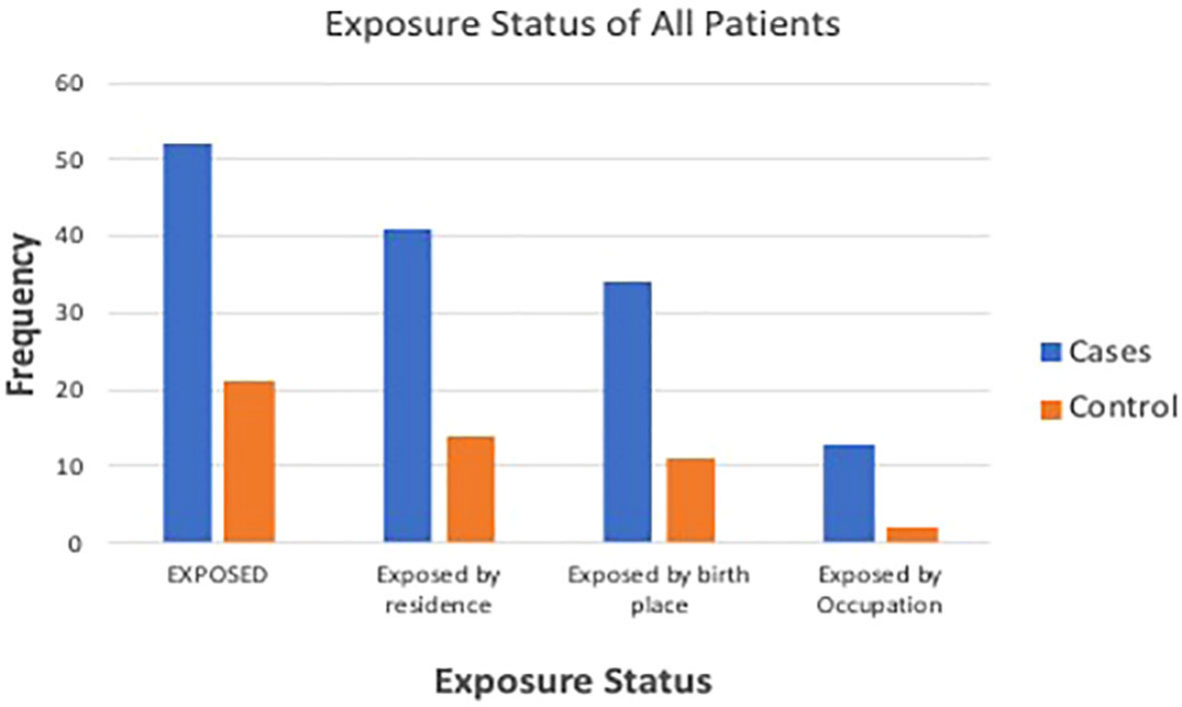
Exposure status of the patients.

There were 52 (70.3%) cases categorized as exposed, compared with 21 (28.4%) controls [OR 5.9 (CI 2.9 to 12.1); *p *< 0.001]. There were 34 (45.9%) cases born near petrochemical plants, compared with 11 (14.9%) controls (*p = *0.001). There were 34 (45.9%) cases residing near petrochemical plants, compared with 8 (10.9%) controls (*p = *0.001). When asked ‘Do you think you have been significantly exposed to crude oil?’, 15 (20.3%) cases answered ‘yes’, compared with only 2 (2.7%) controls (*p* ≤ 0.001). **Figure 1** shows the exposure status of cases and controls. Duration of exposure to petrochemical products was >10 years for 73% of the exposed CKD patients, whereas the remaining 27% had been exposed for 1–10 years (**Table 2**).

**Table 2:** Duration of exposure among the cases.

Duration of exposure (years)	Cases, *n* (%)
5	7 (13.5)
6–10	7 (13.5)
11–20	14 (26.9)
21–30	14 (26.9)
30–40	7 (13.5)
> 40	3 (5.7)
Total	52 (100.0)

### Comparison of traditional CKD risk factors among exposed and less exposed CKD patients

**Table 3** shows the clinical parameters of patients with CKD, according to exposure status. Although a higher percentage of exposed, than less exposed, CKD patients smoked, reported a history of hypertension, diabetes, dyslipidemia, use of nephrotoxins, and a family history of CKD, these differences did not reach statistical significance.

**Table 3:** Comparison of traditional chronic kidney disease (CKD) risk factors among exposed and less exposed CKD patients.

Characteristic	Exposed patients (*N* = 52), *n* (%)	Less exposed patients (*N* = 22), *n* (%)	*p*-value
Male	22 (42.3)	11 (50.0)	0.613
Female	30 (57.7)	11 (50.0)	
Smoking history	22 (43.1)	6 (27.3)	0.295
History of hypertension	25 (48.1)	8 (36.4)	0.446
History of dyslipidemia	3 (5.8)	0 (0.0)	0.550
Family history of kidney disease	3 (5.8)	0 (0.0)	0.552
History of sore throat	2 (3.8)	0 (0.0)	1.000
Use of herbal remedies	3 (5.8)	1 (4.5)	1.000
Use of skin lighteners	3 (5.8)	4 (5.4)	1.000
Use of NSAIDs	10 (19.2)	4 (5.4)	0.491
Use of herbicides/pesticides	9 (17.3)	1 (4.5)	0.264
**Clinical and laboratory measurement**	**Mean ± SD**	**Mean ± SD**	
Age (years)	32.8 ± 8.6	26.7 ± 7.9	**0.005**
BMI (kg/m^2^)	24.5 ± 4.4	22.6 ± 4.6	0.115
Waist circumference (cm)	88.0 ± 11.0	81.5 ± 11.4	**0.025**
SBP (mmHg)	125.8 ± 20.1	127.1 ± 24.1	0.810
DBP (mmHg)	80.3 ± 17.1	77.0 ± 12.3	0.418
Random blood sugar level (mg/dl)	107.1 ± 24.0	95.5 ± 10.6	**0.034**

BMI, body mass index; DBP, diastolic blood pressure; NSAID, non-steroidal anti-inflammatory drug; SBP, systolic blood pressure.

The mean age of exposed CKD patients was higher than that of less exposed CKD patients (*p = *0.005), and, similarly, mean waist circumference was higher in exposed CKD patients than in less exposed CKD patients (*p *= 0.025). Random blood sugar levels were within normal limits among less exposed CKD patients, but were significantly higher among exposed patients (*p = *0.034). Mean SBP and DBP were higher in exposed patients, but the differences did not reach statistical significance. Among controls, mean SBP and DBP were significantly higher in less exposed patients (117.7 ± 8.3 mmHg and 76.5 ± 6.0 mmHg, respectively; *p = *0.026) than in exposed patients (112.5 ± 10.5 mmHg and 71.3 ± 10.0 mmHg, respectively; *p = *0.024), although all values were within normal ranges. There was no difference regarding the frequency of hypertension among exposed and less exposed controls (4.8% and 3.8%, respectively; *p = *1.000). The use of skin lighteners and herbicides/pesticides was significantly higher in exposed controls than in less exposed controls. Although 14.8% of exposed controls used skin lighteners, no less exposed controls used skin lighteners (*p = *0.021). Similarly, 28.6% of exposed controls used pesticides/herbicides, compared with 3.8% of less exposed controls (*p = *0.005).

## Discussion

The authors assessed environmental exposures and locational differences between patients with suspected CGN (i.e., cases) and non-CKD controls residing in the Niger Delta region of Nigeria. Subjects in this case–control study were well matched with regard to age and sex, with the aim of reducing any bias in determining the effect of putative environmental factors such as hydrocarbon exposure. A significantly higher proportion of cases than of controls self-reported being exposed to petrochemical products, and this was seen across categories of current residence, place of birth, and occupation. In addition, cases who were exposed had a significantly higher mean age, mean waist circumference, and blood sugar level (although within normal limits) than less exposed cases.

Our findings suggest that chronic exposure to hydrocarbons may be linked with the development of CKD; however, this should not be interpreted as evidence for causality. Other researchers have reported similar findings, although some researchers had contradictory findings (3). Again, a relatively higher environmental health risk awareness among persons living in oil-producing areas might have biased their responses (18). A comparative cross-sectional study carried out in similar oil-producing areas among petrol station attendants and suitably matched controls reported a significant increase in levels of serum creatinine among the petrol pump workers (19), and this suggests a role for occupational exposure to petrochemicals in reduced kidney function. Similarly, in a nested case–control study, researchers reported an increased cancer risk among oil refinery workers in Finland (20); however, the cross-sectional study of petrol station attendants by Abou-ElWafa et al. showed no significant difference in renal function between such groups (21). Another cross-sectional study, conducted in Niger Delta, Nigeria (i.e., the same area as the current study), found a higher prevalence of CKD in oil-producing areas than in other parts of the country, thus highlighting geographical differences in prevalence and the effect of environmental factors in disease causation. Although these studies suggest a possible hydrocarbon-associated CKD, it is noteworthy that the studies were mainly cross-sectional studies, which are relatively weak in establishing causality. Longitudinal studies or experimental studies are preferable, but the long latency of CKD and toxicity of hydrocarbons, respectively, are barriers to implementing such studies. Case–control designs, like the present study, can be useful.

Another observation in this study was that exposed patients had a higher mean age than less exposed patients, and this may be related to the long latent period between environmental exposure and the manifestation of chronic disease. Similarly, the fact that mean waist circumference was higher in exposed cases than in less exposed cases may be explained by the trend of increasing abdominal fat with increasing age, usually due to a reduction in physical activity with age, among other reasons (22). As expected, hypertension was more common among CKD patients than among controls, and, similarly, mean SBP, urea, and creatinine were higher. The mean body mass index (BMI) was higher among cases, suggesting obesity, which, again, is not surprising, as CKD has been associated with increased metabolic risks. However, more important is the fact that fluid retention in some CKD patients could have contributed to the elevated BMI, drawing attention to the unreliability of this measure in determining body fat composition, especially in CKD patients.

The use of non-steroidal anti-inflammatory drugs (NSAIDs) and herbicides/pesticides was proportionally higher in exposed cases than in less exposed cases, although this finding was not statistically significant. The long-term use of NSAIDs and these chemical substances is common to farmers. However, the long-term use of NSAIDs and herbicides/pesticides is known to have a deleterious effect on the kidneys (4, 23, 24), and with concurrent hydrocarbon exposure this may lead to a synergistic harmful effect on the kidneys, as has been proposed in the theory of multi-causality (25). Further research in the study setting might reveal if farmers are at higher risk of CKD than other less exposed workers. Further research will also be important to investigate whether or not there is a geographical clustering in farmers operating in oil-producing areas with regard to chronic kidney disease, thereby exploring the intersection of environmental and occupational exposures to hydrocarbons/organophosphate chemicals. The findings from such research could potentially contribute to strategies and policies to mitigate environmental health risks.

The main limitation of this study was the use of self-reported residential proximity to petrochemical plants or refineries and of occupational history as a proxy for exposure, rather than the actual measurement of hydrocarbons either in the environment or in participants, and, for this reason, health risk could not be estimated. Although real-time measurement of exposures is a more valid, accurate, and reliable assessment of exposure, the authors note that hydrocarbons are present in multiple forms, from point and non-point sources, and exposure can be through multiple pathways, making it difficult to isolate any one form or pathway as responsible for observed health risk. In addition, the presence of hydrocarbons in biological samples (e.g., urine or blood) from patients does not invariably reflect chronicity of exposure or causation. The authors assessed all residential history, occupational history, and other relevant exposure, and compared this with available hospital records to improve validity and reliability. In addition, a minimum duration of 5 years’ residence in an exposed area was an inclusion criterion to strengthen the argument for chronicity of exposure.

## Conclusion

Our findings suggest an association between exposure to petrochemical products and CKD in young Nigerians living in the Delta State diagnosed with suspected CGN. Apart from a relatively higher mean age, waist circumference, and blood sugar level among the exposed patients, traditional environmental risk factors for CKD were not significantly more prevalent in the exposed patients than in less exposed patients, suggesting that exposure to petrochemical products seems to play a role in the cases of CKD in this study.

These findings should prompt more emphasis on occupational history, residential history, and other relevant environmental exposures in the assessment of patients at risk for CKD. Effective and inclusive public education on environmental health risks is recommended to encourage safer and healthier behaviors. Finally, large population-based epidemiological studies, which will employ reliable and valid measurements of (CKD) exposure and outcome, are needed to help determine the extent to which environmental and occupational hydrocarbon exposure contributes to the prevalence of CKD. We recommend heightened environmental surveillance and safety by government and related organizations in the industry to mitigate the effect of petrochemical exposure in the study area.

## Data availability statement

The raw data supporting the conclusions of this article will be made available by the authors, without undue reservation.

## Ethics statement

The studies involving human participants were reviewed and approved by the Health and Research Ethics Committee, Delta State University Teaching Hospital, Oghara. The patients/participants provided their written informed consent to participate in this study.

## Author contributions

Conceptualization: OO; data curation: OO; formal analysis: OO; methodology: OO and NA; writing original draft: OO and NA; and writing, reviewing and editing: OO and NA. All authors contributed to the article and approved the submitted version.

## References

[1] Pruss-Ustun A Wolf J Corvalan C Bos R Neira M. Preventing disease through healthy environments: A global assessment of the burden of disease from environment risks. Geneva, Switzerland: World health organisation (2016).

[2] Department of Public Health Environmental and Social determinants of Health World Health Organisation. Exposure to benzene: a major public health concern. Geneva, Switzerland: World health organisation (2019).

[3] Okoye OC. Environmental exposure to crude oil : a potential risk for chronic kidney disease(CKD) in diasadvantaged countries. West Afr J Med (2019) 36(2):144–57. 31385601

[4] Garcia-Garcia G Jha V World Kidney Day Steering C. Chronic kidney disease in disadvantaged populations: The case of Africa. Afr J Primary Health Care Family Med (2015) 7(1):e1–2. doi: 10.4102/phcfm.v7i1.839 PMC4656927 26245587

[5] Goldstein BD OH Lichtveld MY. The gulf oil spill. New Engl J Med (2011) 364:1334–48. doi: 10.1056/NEJMra1007197 21470011

[6] Orisakwe OE. Crude oil and public health issues in Niger delta, Nigeria: Much ado about the inevitable. Environ Res (2021) 194:110725. doi: 10.1016/j.envres.2021.110725 33428909

[7] Wu C-D Chern Y-R Pan W-C Lung S-CC Yao T-C Tsai H-J. Effects of surrounding environment on incidence of end stage renal disease. Sci Total Env (2020) 723:137915. doi: 10.1016/j.scitotenv.2020.137915 32392675

[8] Okoye OC Carnegie E Mora L. Air pollution and chronic kidney disease risk in oil and gas-situated communities: A systematic review and meta-analysis. Int J Public Health (2022) 67:1604522. doi: 10.3389/ijph.2022.1604522 PMC9035494 35479765

[9] Wu MY Lo WC Chao CT Wu MS Chiang CK. Association between air pollutants and development of chronic kidney disease: A systematic review and meta-analysis. Sci Total Env (2020) 706:135522. doi: 10.1016/j.scitotenv.2019.135522 31864998

[10] Bamber AM Hasanali SH Nair AS Watkins SM Vigil DI Van Dyke M. A systematic review of the epidemiologic literature assessing health outcomes in populations living near oil and natural gas operations: Study quality and future recommendations. Int J Environ Res Public Health (2019) 16(12). doi: 10.3390/ijerph16122123 PMC6616936 31208070

[11] Ravnskov U. Experimental glomerulonephritis induced by hydrocarbon exposure: a systematic review. BMC Nephrol (2005) 6:15. doi: 10.1186/1471-2369-6-15 16354301 PMC1334181

[12] Ravnskov U. Hydrocarbons may worsen renal function in glomerulonephritis a meta-analysis. Am J Ind Med (2000) 37:599–606. doi: 10.1002/(sici)1097-0274(200006)37:6<599::aid-ajim4>3.0.co;2-x 10797503

[13] Ravnskov U. Hydrocarbon exposure may cause glomerulonephritis and worsen renal function: evidence based on hill's criteria for causality. Q J Med (2000) 93:551–6. doi: 10.1093/qjmed/93.8.551 10924538

[14] Poole C Dreyer NA Satterfield MH Levin L Rothman KJ. Kidney cancer and hydrocarbon exposures among petroleum refinery workers. Environ Health Perspectives (1993) 101:53–62. doi: 10.1289/ehp.93101s653 PMC1520011 8020449

[15] World Health Organisation. The WHO STEPwise approach to chronic disease risk factor surveillance (STEPS). Geneva, Switzerland: world health organisation.

[16] National Institute of Health. BTEX questionnaire national institute of health (2012). Available at: https://gulfstudy.nih.gov/en/docs/BTEX%20Questionnaire.pdf.

[17] Yuan T-H Ke D-Y Wang JE-H Chan C-C. Associations between renal functions and exposure of arsenic and polycyclic aromatic hydrocarbon in adults living near a petrochemical complex. Environ pollut (2020) 256. doi: 10.1016/j.envpol.2019.113457 31785941

[18] D'Andrea MA Reddy GK. Health risks associated with crude oil spill exposure. Am J Med (2014) 127(9):886.e9 –13. doi: 10.1016/j.amjmed.2014.04.035 24859637

[19] Festus OO Dada FL Iweka FK Eyaufe AO Osaigie RN Osaigie EV. Plasma renal functions amongst petrol station attendants in owerri, south-east Nigeria. Int J Community Res (2013) 2(2):34–8.

[20] Anttila A Pokhrel A Heikkilä P Viinanen R Pukkala E. Kidney cancer risk in oil refining in Finland: a nested case-referent study. J Occup Environ Med (2015) 57(1):68–72. doi: 10.1097/JOM.0000000000000301 25563542

[21] Abou-ElWafa HS Albadry AA El-Gilany AH Bazeed FB. Some biochemical and hematological parameters among petrol station attendants: A comparative study. BioMed Res Int (2015) 2015:418724. doi: 10.1155/2015/418724 26634207 PMC4655017

[22] Jura M Kozak LP. Obesity and related consequences to ageing. Age (Dordr) (2016) 38(1):23. doi: 10.1007/s11357-016-9884-3 26846415 PMC5005878

[23] Kataria A Trasande L Trachtman H. The effects of environmental chemicals on renal function. Nat Rev Nephrol (2015) 11(10):610–25. doi: 10.1038/nrneph.2015.94 PMC4689732 26100504

[24] Stanifer JW Jing B Tolan S Helmke N Mukerjee R Naicker S. The epidemiology of chronic kidney disease in sub-Saharan Africa: a systematic review and meta-analysis. Lancet Global Health (2014) 2(3):e174–81. doi: 10.1016/S2214-109X(14)70002-6 25102850

[25] Krieger N. Epidemiology and the web of causation: has anyone seen the spider? Soc Sci Med (1994) 39(7):887–903. doi: 10.1016/0277-9536(94)90202-X 7992123

