# Supplementary figures and images for: Is exposure to hydrocarbons associated with chronic kidney disease in young Nigerians? A case–control study
Source: Front Nephrol. 2022 Dec 16;2:1010080. doi: 10.3389/fneph.2022.1010080 (PMC10479625; doi:10.3389/fneph.2022.1010080)

## FLOWCHART OF THE PATIENT RECRUITMENT OVER 6 MONTHS

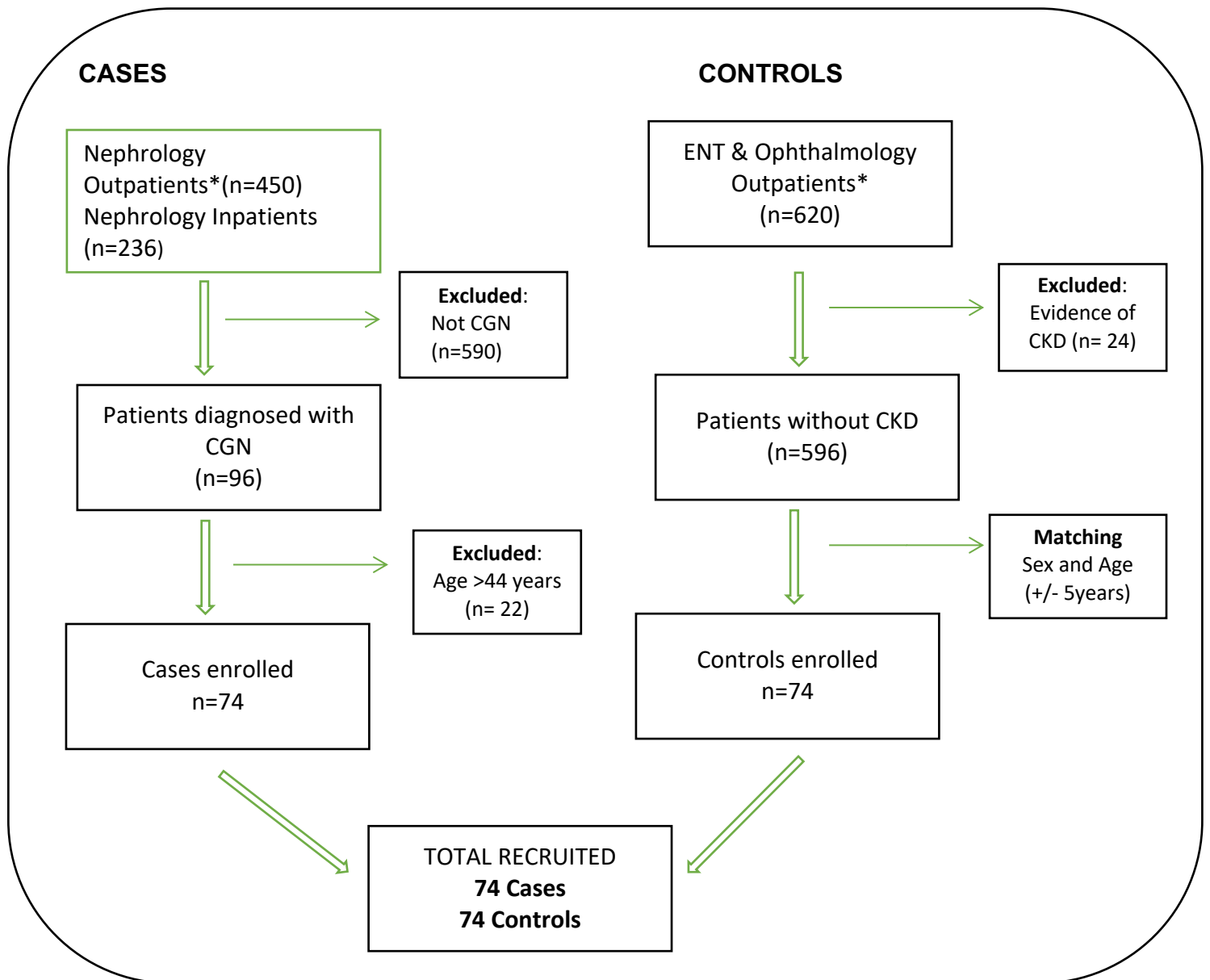

Supplement: Supplementary file 1 [file DataSheet_1.pdf]
